# Comparative Analysis of the Endophytic Bacterial Diversity of *Populus euphratica* Oliv. in Environments of Different Salinity Intensities

**DOI:** 10.1128/spectrum.00500-22

**Published:** 2022-05-19

**Authors:** Haitao Yue, Luyu Zhao, Duo Yang, Minwei Zhang, Jieyi Wu, Zhongkai Zhao, Xiangxiang Xing, Liwen Zhang, Yanan Qin, Fei Guo, Jie Yang, Tuerxunnayi Aili

**Affiliations:** a Department of Bioengineering, School of Life Science and Technology, Xinjiang University, Urumqi, People’s Republic of China; b Laboratory of Synthetic Biology, School of Future Technology, Xinjiang University, Urumqi, People’s Republic of China; c Biotechnology Research Institute, The Chinese Academy of Agricultural Sciences, Beijing, People’s Republic of China; State Key Laboratory of Microbial Resources, Institute of Microbiology, Chinese Academy of Sciences

**Keywords:** *Populus euphratica*, endophytic bacteria, saline environment

## Abstract

Populus euphratica Oliv. has a high tolerance for drought, salinity, and alkalinity. The main purpose of this study is to explore the effects of environments of different salinity intensities on endophytic community structure and the possible roles of endophytes in the tolerance of host plants. The characterization of endogenous bacteria in diversity has been investigated by using the Illumina high-throughput sequencing technique. The research showed that endophytic bacteria of *P*. *euphratica* in an extremely saline environment had low species diversity, especially in sap tissue. The dominant phyla in all groups were *Proteobacteria*, *Actinobacteria*, and *Bacteroidetes*. Notably, *Firmicutes* (relative abundance >5%) was a different dominant phylum in the samples from the high-saline environment compared with the relatively low-saline-environment group. The linear discriminant analysis effect size (LEfSe) analysis found that there were significant differences in different saline environments of *Cytophagaceae* (family), *Rhodobacteraceae* (family), and *Rhodobacterales* (order). These results indicated that the composition of the endogenous bacterial community was related to the growth environment of host plants. The predictive analysis of KEGG pathways and enzymes showed that the abundance of some enzymes and metabolic pathways of endophytes of *P. euphratica* increased with the increase of soil salinity, and most of the enzymes were related to energy metabolism and carbohydrate metabolism. These findings suggested that the endogenous bacteria of the host plant had different expression mechanisms under different degrees of stress, and this mechanism was very obvious in the distribution of endophytes, while the function of the endogenous bacteria needs to be further explored.

**IMPORTANCE** Euphrates poplar (Populus euphratica Oliv.), as the only tree species that grows in the desert, has tenacious vitality with the characteristics of cold tolerance, drought tolerance, salt-alkali tolerance, and wind-sand resistance. *P. euphratica* has a long growth cycle and a high growth rate, which can break wind, fix sand, green the environment, and protect farmland, making it an important afforestation tree species in arid and semiarid areas. The area of *P. euphratica* in Xinjiang accounts for 91.1% of its area in China. Studying the endophytic bacteria of *P. euphratica* can give people a systematic understanding of it and the adaptability of the endogenous flora to the host and special environments. In this study, by analyzing the endophytic bacteria of *P. euphratica* in different saline-alkali regions of Xinjiang, it was found that the bacteria in different tissues of *P. euphratica* changed with the change of soil salinity. Especially in the sap tissue of *P. euphratica* under extremely high salinity, the diversity of endogenous bacteria was significantly lower than that in other tissues. These differential bacteria under different salinities were mostly related to the stress resistance of themselves and the host. Not only that, we also selected a strain of *Bacillus* with high stress resistance from the tissues of *P. euphratica*, which can survive under the extreme conditions of 10% NaCl and pH 11. We obtained a genome completion map of this strain, named it Bacillus haynesii P19 (GenBank accession no. PRJNA648288), and tried to use it for fermentation but in a different work, so as to develop it into a promising industrial fermentation chassis bacterium. Therefore, this study was of great significance for the understanding of endophytic bacteria in *P. euphratica* and the acquisition of extremophilic microbial resources.

## INTRODUCTION

Salt-affected soils (SAS) are a global issue. Data from the Food and Agriculture Organization of the United Nations (FAO) indicates that greater than 424 million hectares of topsoil (0 to 30 cm) and 833 million hectares of subsoil (30 to 100 cm) are salt affected: 85% of salt-damaged topsoils are saline, 10% are sodic, and 5% are saline-sodic, while 62% of salt-affected subsoils are saline, 24% are sodic, and 14% are saline-sodic (1). Among them, more than two-thirds of global SAS are found in arid and semiarid climatic zones, covering five continents except Antarctica: 37% of SAS are located in arid deserts and 27% of SAS are distributed in the arid steppe (half in the cold arid steppe and half in the hot arid steppe). These data also show that 20% to 50% of irrigated soils on all continents are too saline; this means that more than 1.5 billion people in the world are facing major challenges in food production due to soil degradation. Therefore, it is urgent for humankind to prevent soil salinization and improve biodiversity and the ecological environment.

For the prevention and control of SAS, one of the methods is to plant salt-tolerant plants (2). However, due to climate, topography, and landforms, the distribution area of SAS has little precipitation and considerable evaporation. The annual precipitation is not enough to wash away the accumulated salt on the surface of the soil. The accumulation of salt makes the vegetation that can grow very limited. Most plants are herbs and shrubs, and there are extremely few tall trees (3). For example, in Australia, the typical plants of *Eucalyptus* growing in the dry subtropical regions of the southeast and southwest mostly appear as bushes under the influence of geography and climate (4). The characteristic vegetation of the Nevada desert in the United States is the desert scrub Larrea tridentata*-*Ambrosia dumosa (5). Euphrates poplar (Populus euphratica Oliv.), as the only tree species that grows in the desert, has tenacious vitality with the characteristics of cold tolerance, drought tolerance, salt-alkali tolerance, and wind-sand resistance (6). *P. euphratica* has a long growth cycle and a high growth rate, which can break wind, fix sand, green the environment, and protect farmland, making it an important afforestation tree species in arid and semiarid areas (7).

In the past 30 years, the main research on plants in saline-alkali land has focused on the resistance of plants to salt stress. Due to the high adaptability of *P. euphratica* to the extreme desert environment, it has become an important model species for studying the effects of abiotic stresses on trees (8). Research on the molecular biology of *P. euphratica* has made progress in genetic diversity (9) and functional genes, genome, and transcriptomics (10). With the development of microbiology research, researchers found that the plant’s endophytic microorganisms have a close relationship with the host and have a positive impact on the adaptability to the host’s special environment. It has been demonstrated that in areas with abiotic stress factors, plants are more dependent on microorganisms that are able to enhance their ability to combat stress (11).

However, the research on the endogenous bacteria of *P. euphratica* mainly focuses on the isolation and identification of culturable endophytic microorganisms (12) and the performance of single bacteria isolated from *Populus euphratica* (13), while the understanding of the diversity and community structure of its endophytic bacteria is still lacking. In China, *P. euphratica* is primarily distributed in Xinjiang, Inner Mongolia, Qinghai, Gansu, and Ningxia. Among them, the area of *P. euphratica* growth in Xinjiang accounts for 91.1% of its growth in China. Studying the endophytic bacteria of *P. euphratica* can give people a systematic understanding of it and the adaptability of the endogenous flora to the host and special environments.

Therefore, this study analyzed the endophytic bacteria of *P. euphratica* on different saline-alkali soils in Xinjiang, observed whether the endophytic bacterium was stable or changed with the increase of the salt-alkali concentration, and clarified whether the endogenous bacteria of *P. euphratica* were related only to its own saline-alkali environment or would alter with variations in the external environment. If there is a change, is it related to the function of the endophytic bacteria? The findings could provide clues for the systematic recognition of endogenous bacterial groups in *P. euphratica*, especially the response of endophytic bacteria to the changes of salt environment, and the description of the interaction between the environment and the endogenous bacteria of the plant.

## RESULTS AND DISCUSSION

### Soil physicochemical properties of *P. euphratica* growth area.

We collected and detected the physicochemical properties of the soils where *P. euphratica* grows, and the results showed that SO_4_^2−^ and Cl^−^ were the predominant anions in the soils from both areas. In addition, the main cations of the soils in Zephyr County were Na^+^ and K^+^, while the soils from the Darbancheng District had an individually high content of Ca^2+^ in addition to Na^+^ and K^+^. The pH values of soil samples ZP_8 and ZP_9 collected from Zephyr County were 9.11 and 8.81, respectively, and the electrical conductivity (ECe) values were 10.11 dS/m and 18.95 dS/m, respectively, while the pH values of soil samples DBC_8 and DBC_9 collected from the Darbancheng District were 7.13 and 7.09 and the ECe values were 3.69 dS/m and 6.58 dS/m, respectively (Table 1).

**TABLE 1:** Soil physicochemical properties of soil samples

Soil physicochemical property	Value for soil type and sample			
	S_saline, ZP_9	H_saline, ZP_8	M_saline, DBC_9	L_saline, DBC_8
Content (g/kg)				
CO_3_^2−^	0.061	0.014	0.000	0.000
HCO_3_^−^	0.366	0.202	0.328	0.236
Cl^−^	9.208	3.656	2.010	0.937
Ca^2+^	0.600	1.350	4.340	2.670
Mg^2+^	5.835	0.838	0.525	0.262
SO_4_^2−^	7.685	1.567	2.683	2.016
K^+^	0.523	8.049	0.158	0.129
Na^+^	3.486	1.236	1.252	0.559
pH	8.810	9.110	7.090	7.130
ECe (dS/m)	18.950	10.110	6.580	3.690
Salinity intensity (FAO)	Extreme	Very strong	Robust	Moderate

The FAO considers soils with ECe values of >2 dS/m and pH >8.2 to be SAS. According to this standard, the soil samples we collected all belonged to SAS. Among them, an ECe value of >15 is extreme salinity intensity, and the ECe values between 8 and 15 are very strong salinity intensity. The ECe values between 4 and 8 are robust salinity intensity, and the ECe values between 2 and 4 are moderate salinity intensity. Therefore, we grouped ZP_9, ZP_8, DBC_9, and DBC_8 into S_saline, H_saline, M_saline, and L_saline. Since *P. euphratica* grows poorly in a hot, moist climate and heavy clay soil, it mostly lives in a dry climate (14). We did not observe the growth of *P. euphratica* in healthy soil under the natural environment of Xinjiang, so we could compare the differences in the endophytic microbial community of *P. euphratica* only under various salinities. There was no way to use *P. euphratica* grown in nonsaline soil as a control.

### Alpha diversity of the microbial community.

In order to understand the endophytic microbial community of *P. euphratica* in different saline environments, we analyzed the alpha diversity index of each sample by MiSeq high-throughput sequencing, including the read numbers, coverage, Shannon index, and Chao index of each sample at a genetic distance of 3% (Table 2). The samples collected from the *P. euphratica* forest can be divided into three types: sap, xylem, and soil. Through alpha diversity analysis, we can get the relevant index reflecting the diversity and richness of the microbial community in the samples. Quality and chimera filtration of the raw data produced in total 680,897 high-quality sequencing Reads, ranging from 10,681 bp to 61,392 bp. The coverage values of all samples were greater than 0.99, which indicated that the sequencing depth was sufficient to cover most microorganisms.

**TABLE 2:** Alpha diversity index of each sample of *P. euphratica*

Group	Sample type	Sample identifier	No. of reads	Coverage	Alpha diversity	
					Shannon	Chao
S_saline						
S_saline_sap	Sap	ZP_1	10,681	0.99737	2.69762	140.0000
		ZP_2	17,325	0.9974	2.79639	230.6666
S_saline_xylem	Xylem	ZP_4	31,909	0.99511	4.71727	927.9285
		ZP_5	32,844	0.99558	4.94794	815.3170
S_saline_soil	Soil	ZP_9	47,914	0.99647	5.36877	1,295.3050

H_saline						
H_saline_sap	Sap	ZP_3	46,217	0.99645	4.44534	922.3589
H_saline_xylem	Xylem	ZP_6	44,580	0.99748	3.84021	573.5384
		ZP_7	51,985	0.9978	4.27084	745.7205
H_saline_soil	Soil	ZP_8	45,586	0.99618	4.55783	855.5816

M_saline						
M_saline_sap	Sap	DBC_1	35,509	0.99659	4.76852	691.4375
M_saline_xylem	Xylem	DBC_5	26,235	0.99733	4.54128	491.5660
M_saline_soil	Soil	DBC_9	32,787	0.99594	6.00210	1,387.0720

L_saline						
L_saline_sap	Sap	DBC_2	29,563	0.99644	4.31679	615.2222
		DBC_3	45,454	0.9967	4.91200	944.8829
L_saline_xylem	Xylem	DBC_4	36,439	0.99596	4.91057	793.8658
		DBC_6	61,392	0.99747	5.14457	1,062.0670
		DBC_7	42,884	0.99706	4.92196	749.8088
L_saline_soil	Soil	DBC_8	41,593	0.99689	4.89385	844.0153

The Chao index primarily reflects the number of operational taxonomic unit (OTU) species, while the Shannon index is relative to OTU diversity. The results showed that the Shannon index and Chao index of the sap tissue of *P. euphratica* under extremely high salinity were modest compared with other groups, indicating that its microbial number and diversity were both lower. Similar results were also found in saline samples from the Arava Valley, of which the microbial diversity was limited because of the extreme habitats (15). However, the Shannon and Chao indexes of xylem and soil samples of *P. euphratica* under different salinities did not differ greatly. Therefore, we can speculate that when the soil salinity is too high, it will affect the diversity of the endogenous bacteria of the sap tissue of *P. euphratica* but will not have much impact on the xylem.

### Microbial community composition.

**FIG 1:**
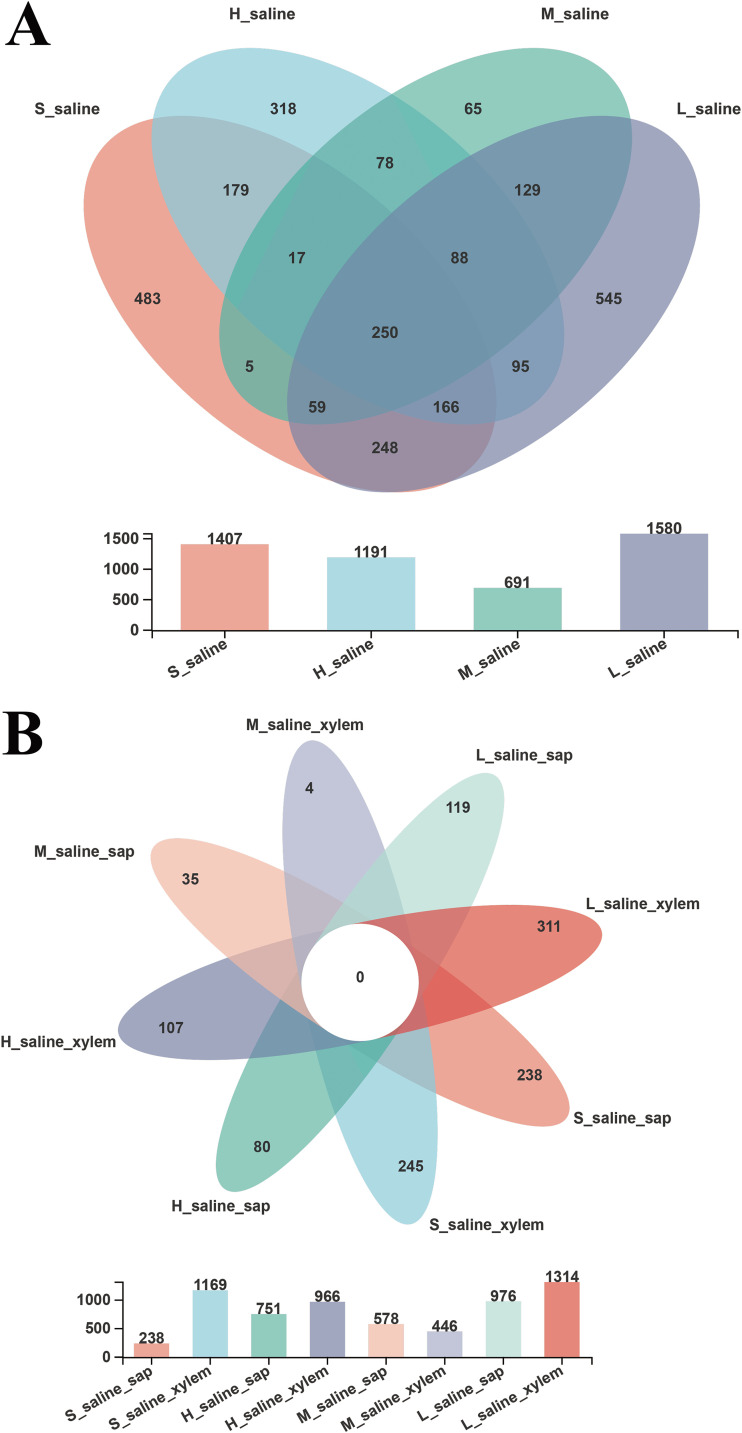
Comparison of OTUs of *P. euphratica* under different salinities (A) and from different tissues (B) by Venn diagram (sequence similarity greater than 97% is classified as an OTU).

For further investigation of the composition of endophytic bacteria in each tissue sample of *P. euphratica* under different salinities, we constructed a Venn diagram to identify the number of OTUs presented in these groups (Fig. 1). Figure 1A showed that the numbers of group-specific OTUs of each group were 483, 318, 65, and 545 according to soil salinity from high to low. There were 250 shared OTUs in the four groups under different salinities, accounting for 5.13% of the total OTUs. These shared OTUs were composed of a number of bacterial groups, including *Firmicutes*, *Proteobacteria*, *Chloroflexi*, *Actinobacteria*, and *Bacteroidetes*. Previous studies also have shown that the majority of the isolated strains belonged to *Firmicutes*, *Proteobacteria*, and *Actinobacteria* (12). For the different tissues of *P. euphratica* under various salinities (Fig. 1B), the sap tissue of *P. euphratica* under extremely high salinity had the fewest OTUs (218), which was consistent with the results of alpha diversity analysis.

**FIG 2:**
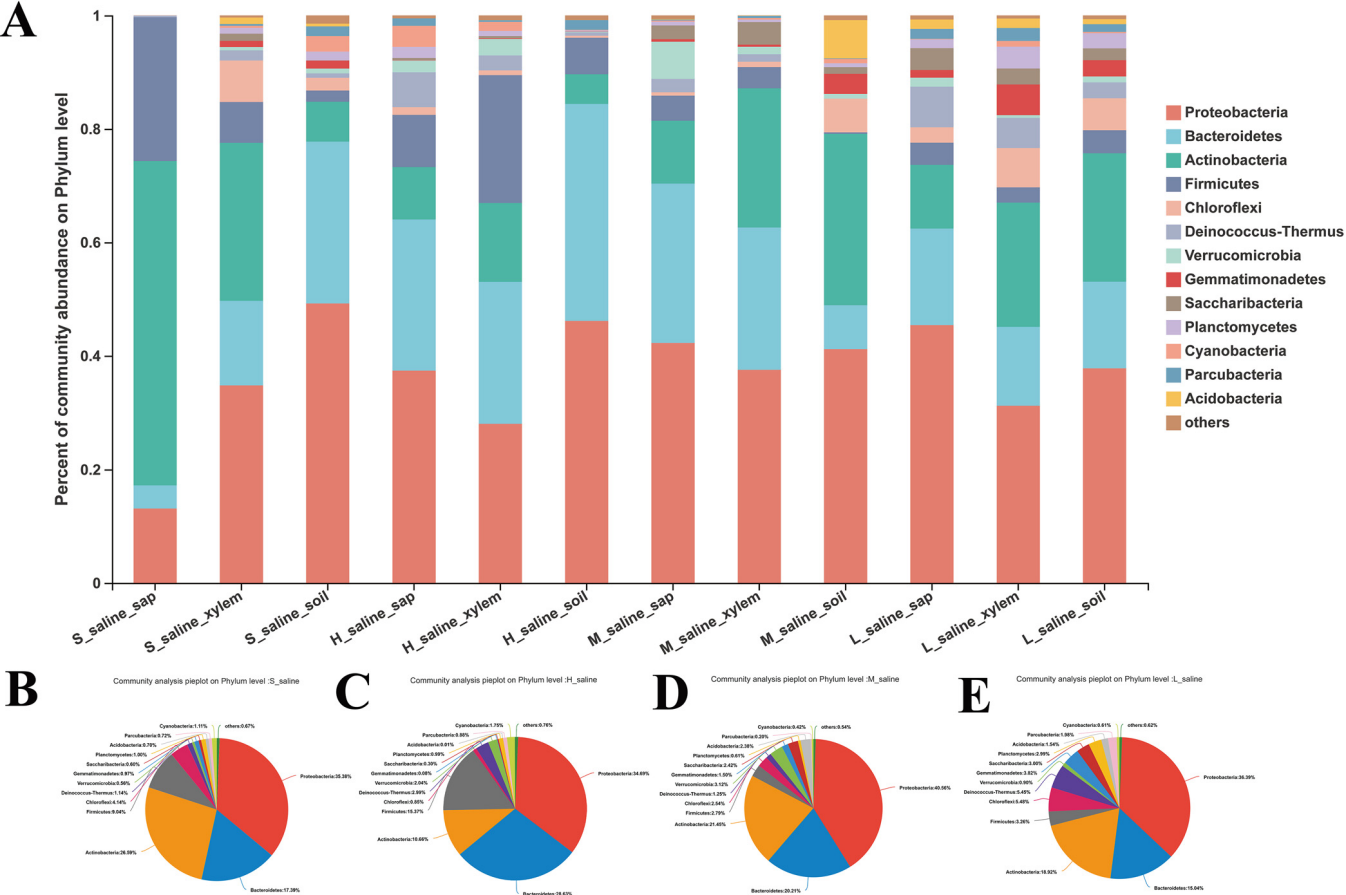
Microbiota composition of different tissues (A) and different salinities (B to E) of *P. euphratica* at phylum level (the phyla that account for less than 1% of the abundance in the sample are merged into “others”).

Then, we clustered these OTUs at the phylum level with a 70% threshold. The results were presented by bar and pie charts. All OTUs were identified as 37 prokaryotic phyla. Figure 2 showed the community composition at the phylum level in varied tissues (Fig. 2A) and different salinities (Fig. 2B to E) of *P. euphratica* (combining the species that account for less than 1% of the abundance in the sample as “others”).

From the bar chart (Fig. 2A), we can see that the endophytes of *P. euphratica* were mainly concentrated in 13 phyla including *Proteobacteria*, *Bacteroidetes*, *Actinobacteria*, *Firmicutes*, *Chloroflexi*, *Deinococcus*-*Thermus*, *Verrucomicrobia*, *Gemmatimonadetes*, *Saccharibacteria*, *Planctomycetes*, *Cyanobacteria*, *Parcubacteria*, and *Acidobacteria*. Among them, *Proteobacteria* was the most abundant phylum across all tissues, and the relative abundance ranged from 13.12% to 49.22%. Most of the *Proteobacteria* in nature are Gram-negative bacteria. They are widely distributed and are important mediators of the nitrogen, sulfur, and carbon cycle in the ecosystem (16). The other two dominant phyla were *Actinobacteria* and *Bacteroidetes*. The relative abundance of the former ranged from 5.24% to 51%, and that of the latter ranged from 4.09% to 38.26%. *Actinomycetes* are known to colonize the internal tissues of plants and play a role in pathogen defense and growth regulation of their hosts (17). The *Bacteroidetes* are very diverse, and environmental *Bacteroidetes* can degrade complex organic matter (18).

It is worth noting that *Firmicutes* was another dominant phylum in the tissues of *P. euphratica*, and the relative abundance ranged from 0.25% to 25.36% (Fig. 2A). The relative abundances of the *Firmicutes* in the four groups under various salinities were quite different, 9.04% and 15.37% in the extreme saline group and the very robust saline group (Fig. 2B and C) and 2.79% and 3.26% in the robust saline group and the moderate saline group (Fig. 2D and E). Formation of endospores is a specific property of *Firmicutes*, which can resist excessive conditions by producing these spores (19). From the higher abundance of *Firmicutes*, it can be inferred that the high-saline environment will affect the composition of the endophytic microbiota of *P. euphratica*, and the *Firmicutes* with high stress resistance can survive to a greater degree in a high-pH and high-salt environment, so that *P. euphratica* can survive better under these conditions, too.

In previous research in the laboratory, 44 strains of cultivable endophytic bacteria were screened and identified from the tissue of *P. euphratica* by using LB medium with a salt content of 3%, and they belonged to 5 genera, namely, *Bacillus*, *Planococcus*, *Nesterenkonia* of *Firmicutes*, *Oerskovia* of *Actinobacteria*, and *Halomonas* of *Proteobacteria*. Among them, the number of cultivable bacteria belonging to *Bacillus* was the largest, accounting for 65.9% of the total. Not only that, a strain of *Bacillus* with strong saline and alkali tolerance was screened from the LB selective medium with high salinity and alkalinity which can survive under the extreme conditions of 10% NaCl and pH 11. We also obtained a genome completion map of this strain, named it Bacillus haynesii P19 (GenBank accession no. PRJNA648288), and tried to use it for fermentation but in a different work (see Fig. 6), so as to develop it into a promising industrial fermentation chassis bacterium (20). Therefore, we speculated that the *Firmicutes* containing *Bacillus* were of great significance for the endophytes of *P. euphratica* and the host’s resistance to a saline-alkali environment.

There was also a special phylum, *Deinococcus*-*Thermus*, in the tissues of *P. euphratica*. The comparative abundances of this phylum in the extreme saline group and very strong saline group were 1.14% and 2.99%, respectively (Fig. 2B and C), while those in the robust saline group and the moderate saline group were 1.25% and 5.45%, respectively (Fig. 2D and E). Moreover, its relative abundance was the highest in the sap and xylem of the moderate saline group of *P. euphratica*, reaching 7.15% and 5.32%, respectively (Fig. 2A). *Deinococcus-Thermus* is recognized as one of the most extremophilic phyla of bacteria and is divided into the orders *Deinococcales* and *Thermales* (21). Sayeh et al. obtained an enriched culture of *Deinococcus-Thermus* from Tunisian geothermal springs in the temperature range of 50 to 75°C (22). *Deinococcus-Thermus* are not only resistant to high temperature and drought but also resistant to radiation. Chanal et al. exposed a sand sample to 15 kGy in a ^60^Co source from the desert of Tataouine and obtained two radiation-tolerant isolates, which were affiliated with the *Thermus-Deinococcus* phylum (23). The high abundance of *Deinococcus-Thermus* in the tissues of *P. euphratica* in semiarid areas with moderate salinity indicated that this phylum was drought tolerant but not saline tolerant.

**FIG 3:**
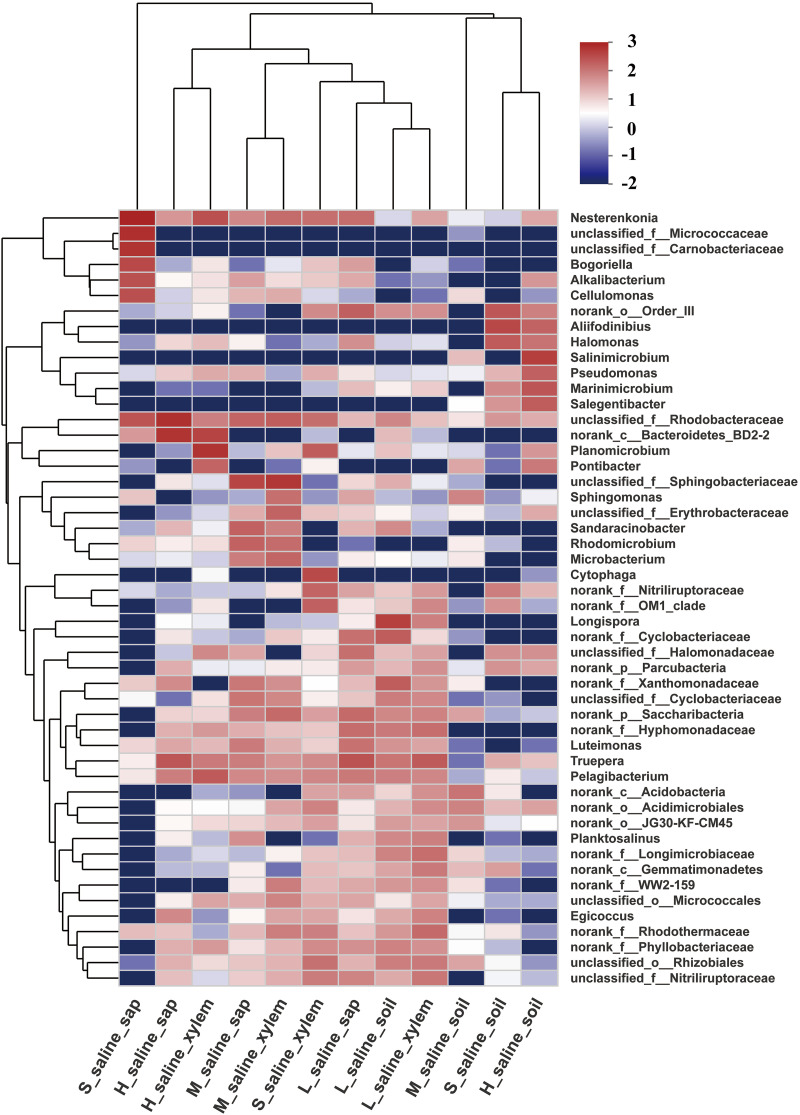
Heatmap of selected most differentially abundant features at the genus level. The top 50 classified genera and 12 sample types were both hierarchically clustered based on Bray-Curtis similarity index.

For more details, the hierarchical clustering heatmap showed the distribution of microbial communities at the genus level in different tissues and under different salinities (Fig. 3). The top 50 classified genera and 12 sample types were both hierarchically clustered based on the Bray-Curtis similarity index. Cluster analysis showed that endophytic flora of the tissues of *P. euphratica* under the same salinity had a correlation. The dominant genera of sap of *P. euphratica* under extreme salinity were very different from those of other groups, including *Nesterenkonia*, unclassified_f_*Micrococcaceae*, unclassified_f_*Carnobacteriaceae*, *Bogoriella*, *Alkalibacterium*, and *Cellulomonas*. Among them, *Nesterenkonia* is a genus of *Actinobacteria*, *Micrococcineae*, and *Micrococcaceae*. The strains of this genus are aerobic, mesophilic, moderately halophilic, or halotolerant. Some species are also alkaliphilic or alkali tolerant (24). Strains have been isolated not only from some extreme environments, such as soda lakes (25), saline and alkaline soils (26), a hypersaline lake (27), Antarctic soil (28), desert soil (29), and so on, but also from human beings (30). Among the 44 strains of cultivable endophytic bacteria screened above, there were also bacteria of *Nesterenkonia* (20). Groth et al. isolated a new alkaliphilic actinomycete of the genus *Bogoriella* from a soda lake in Africa (31). *Cellulomonas* in sap of *P. euphratica* under extreme salinity can produce xylanase to degrade cellulose (32).

**FIG 4:**
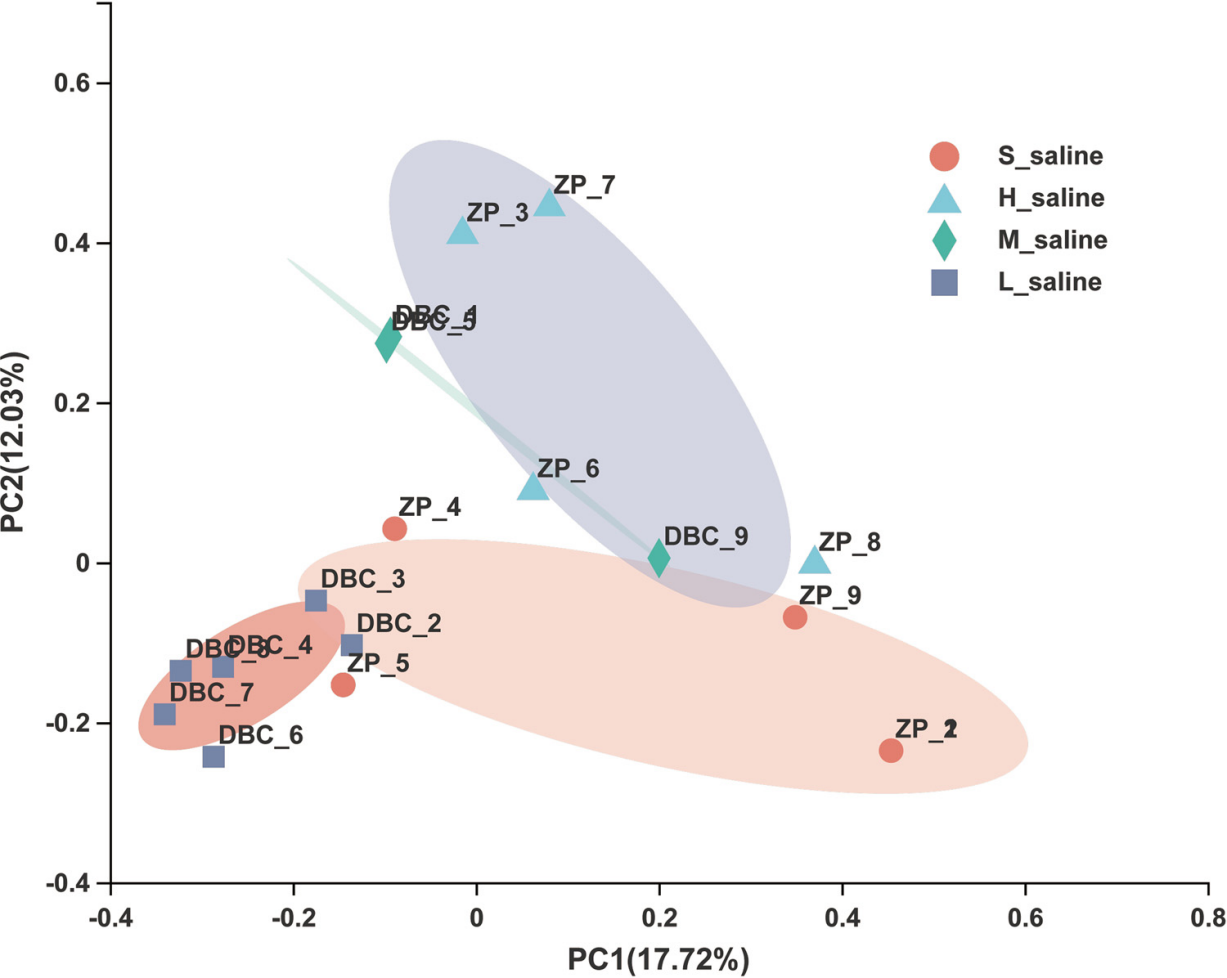
Principal-coordinate analysis (PCoA) using Bray-Curtis metric distances of beta diversity in the samples of *P. euphratica* from different salinities at the OTU level.

It can also be seen from the principal-coordinate analysis (PCoA) results that the microbial community of *P. euphratica* under different salinities had an obvious trend of separation, indicating that with the change of salinity of soil, the microbial community will change accordingly (Fig. 4). The separation trend of sap samples ZP_1 and ZP_2 of *P. euphratica* under extreme salinity compared with other samples was very obvious, indicating that the community composition structure was very different, and the same was true for the alpha diversity (Table 2) and community composition at the phylum level (Fig. 2A) and the genus level (Fig. 3). This may be due to the special growth environment of *P. euphratica*. In order to adapt to the high-saline-alkali environment, *P. euphratica* removes the excess salt absorbed in the soil into white crystals on the trunk or branches to maintain its own osmotic balance. Therefore, the salinity of the sap of *P. euphratica* is relatively high, resulting in a great difference between the microbial community structure of the sap and that of other tissues.

However, although the diversity of the tissue of *P. euphratica* was reduced under extremely high salinity, most of the surviving bacteria were associated with salinity tolerance. As shown in Fig. 3, *Nesterenkonia* and *Bogoriella*, which were highly abundant under extremely high salinity, had been documented to be related to salt tolerance. Culturable bacteria such as *Bacillus* and *Halomonas* screened from the tissue of *P. euphratica* in the early stage of the laboratory work also had literature showing that they can improve the salt tolerance of plants as plant growth-promoting rhizobacteria (33). For example, Upadhyay and Singh found that the inoculation of Bacillus aquimaris could increase the N content of wheat leaves under salt stress (34). Mahgoub et al. applied salt-tolerant endophytic bacteria Bacillus subtilis and Bacillus thuringiensis isolated from halophytes to broad beans under salt stress and found that the bacteria could induce salinity tolerance, enhance nutrient uptake, and promote broad bean growth under salinity stress (35). Zhang et al. treated cotton seeds with rhizosphere salt-tolerant growth-promoting bacteria including *Bacillus* and *Halomonas*, which were isolated from Kalidium foliatum, and effectively improved the growth of cotton under saline-alkali stress (36). Therefore, based on the data of this study and combined with the reported data, we support the idea that endophytes with low diversity can still improve the salinity tolerance of *P. euphratica* under extremely high salinity.

### Significant differences in microbial communities.

**FIG 5:**
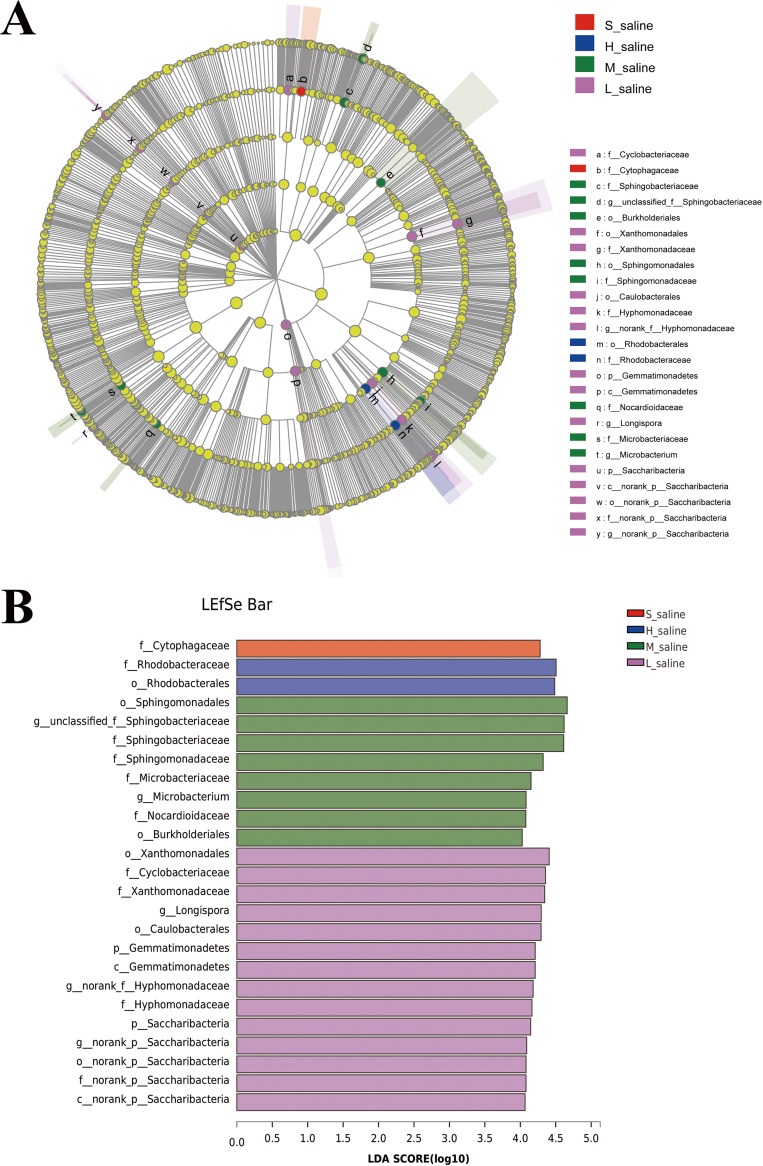
LEfSe analysis of microbial abundance of samples under different salinities. (A) Cladogram of microbial communities. (B) LDA score identified the size of differentiation between saline and mild saline with a threshold value of 4.

To determine the classified bacterial taxa with significant divergences in abundance between different groups under different salinities, we used the linear discriminant analysis (LDA) effect size (LEfSe) method to analyze biomarkers. As shown in Fig. 5A, there were 25 bacterial clades that were statistically significantly different with an LDA threshold of 4 (Fig. 5B). The highest number of bacteria were significantly enriched in the groups of robust and moderate salinity, while only 1 clade showed the advantage of abundance in the group under extreme salinity, which was *Cytophagaceae* (family). Chaudhary et al. isolated two novel psychrotolerant members of the family *Cytophagaceae* from Arctic soil (37). Some researchers have pointed out that the *Cytophagaceae* are widely present in soil, fresh water, and oceans and are the main degraders of cellulosic polysaccharides (38). Therefore, we can use low-cost raw materials containing cellulosic polysaccharides to ferment the strains of this family so as to reduce the cost of industrial production. At the same time, this family is resistant to not only cold but also drought and extreme salinity and has high robustness to maintain its own characteristics without weakening, so that it can better grow in the fermentation system. There were 2 clades that showed the advantage of abundance in the group under very strong salinity, which were *Rhodobacteraceae* (family) and *Rhodobacterales* (order). In previous studies, most *Rhodobacterales* originate from marine habitats but some originate from (hyper-)saline lakes or soil (39).

The above results suggested that different salinity will affect the composition of the endogenous microbial community of *P. euphratica*. Some stress-resistant bacteria in *P. euphratica* can better help it resist the external stress environment. For example, the highly abundant *Firmicutes* of *P. euphratica* under high salinity can help it resist extreme conditions by producing endospores. The high-abundance *Deinococcus-Thermus* in tissues of *P. euphratica* with moderate salinity are not salt tolerant but are tolerant of drought, high temperature, and radiation. Some salt-tolerant or alkali-tolerant bacteria such as *Nesterenkonia*, *Bogoriella*, *Alkalibacterium*, *Cellulomonas*, and *Cytophagaceae* that exist in tissues of *P. euphratica* under extreme salinity will have potential application value if they can be used well.

### Functional prediction of the microbiota.

Since the endogenous bacterial community of *P. euphratica* would change with the change of environmental salinity, we used the PICRUSt2 (Phylogenetic Investigation of Communities by Reconstruction of Unobserved States) tool to predict the function of endophytic bacteria of *P. euphratica* and compared the microbial metabolic functional abundances in different salinity groups.

Based on the results, we can see that the abundance of some predicted enzymes and metabolic pathways of endophytes of *P. euphratica* changed with the change of soil salinity (Tables 3 and 4). These enzymes that were upregulated with increasing salinity were involved in microbial metabolism in diverse environments, such as 3-oxoadipate coenzyme A (CoA)-transferase, phenol 2-monooxygenase, and rhamnulose-1-phosphate aldolase. Mannitol-1-phosphate 5-dehydrogenase and rhamnulose-1-phosphate aldolase were involved in the metabolism of fructose and mannose. There were also enzymes involved in amino acid metabolism such as putrescine oxidase and choline oxidase. In particular, amino acid metabolism and sugar metabolism were very important for endophytic bacteria and the host to resist the external stress environment.

**TABLE 3:** Abundances of enzymes in different salinity groups

Enzyme	Description	Abundance			
		S_saline	H_saline	M_saline	L_saline
2.8.3.6	3-Oxoadipate CoA-transferase	6,580.22	5,828.69	4,821.95	4,703.5
1.1.1.17	Mannitol-1-phosphate 5-dehydrogenase	4,179.15	2,651.65	1,661.91	1,453.26
1.1.1.306	*S*-(Hydroxymethyl)mycothiol dehydrogenase	3,463.98	3,187.88	3,056.98	1,950.35
1.8.1.15	Mycothione reductase	2,753.88	2,171.89	1,987.81	1,188.31
1.14.13.7	Phenol 2-monooxygenase	2,713.43	2,663.21	2,122.94	1,123.9
2.8.4.2	Arsenate-mycothiol transferase	2,658.63	2,006.08	1,015.02	662.22
1.4.3.10	Putrescine oxidase	2,636.6	2,128.5	2,037.33	886.65
4.1.2.19	Rhamnulose-1-phosphate aldolase	2,599.36	1,951.45	1,267.72	900.38
1.1.3.17	Choline oxidase	2,537	1,749.25	884.01	612.83
4.1.2.40	Tagatose-bisphosphate aldolase	2,398.51	1,228.64	1,141.33	531.6

**TABLE 4:** Abundance of KEGG pathways in different salinity groups

Pathway level 1	Pathway level 2	Pathway level 3	Description	Abundance			
				S_saline	H_saline	M_saline	L_saline
Genetic information processing	Transcription	ko03022	Basal transcription factors	4,580.44	4,444.06	4,238.44	2,539.97
Metabolism	Metabolism of terpenoids and polyketides	ko00253	Tetracycline biosynthesis	1,046.96	887.62	537.79	107.78

For example, 3-oxoadipate CoA-transferase can oxidize 3-oxoadipate to 3-oxoadipate-CoA and then through acetyl-CoA C-acyltransferase and 3-oxoadipyl-CoA thiolase to generate succinyl-CoA, which participates in the tricarboxylic acid cycle and provides significant energy for bacteria and plants to resist saline stress. Similarly, mannitol-1-phosphate 5-dehydrogenase is involved in the conversion process between fructose and mannose. The degradation of fructose and mannose is also an important part of glycolysis. Glycolysis can transfer the released free energy to ATP to provide the body with the energy needed for life activities. Phenol 2-monooxygenase can convert phenol to catechol and participate in benzoate degradation. The produced catechol can finally produce succinyl-CoA and acetyl-CoA through a series of degradations and enter the tricarboxylic acid cycle (40). Rhamnulose-1-phosphate aldolase can generate glycolaldehyde, and glycolaldehyde is widely present in the solvent system of organisms. It can react with acrolein to generate ribose, which is very important for the synthesis of RNA in organisms. Rhamnulose-1-phosphate aldolase also participates in fructose and mannose metabolism, which is an important part of glycolysis. Tagatose-bisphosphate aldolase participates in the pentose phosphate pathway and is also one of the main pathways of carbohydrate metabolism in the body. In addition, putrescine oxidase can catalyze the production of putrescine. Putrescine is found in Gram-negative bacteria or fungi and exists in different species in high concentrations. Putrescine is the precursor of spermidine and spermine metabolism. It can control cell pH by increasing or decreasing its content. Therefore, putrescine plays an important role in microbial metabolism. These enzymes highly expressed in a saline environment play a very important role in the resistance of endophytes and plants to salt stress.

### Salt-alkali tolerance of Bacillus haynesii P19.

**FIG 6:**
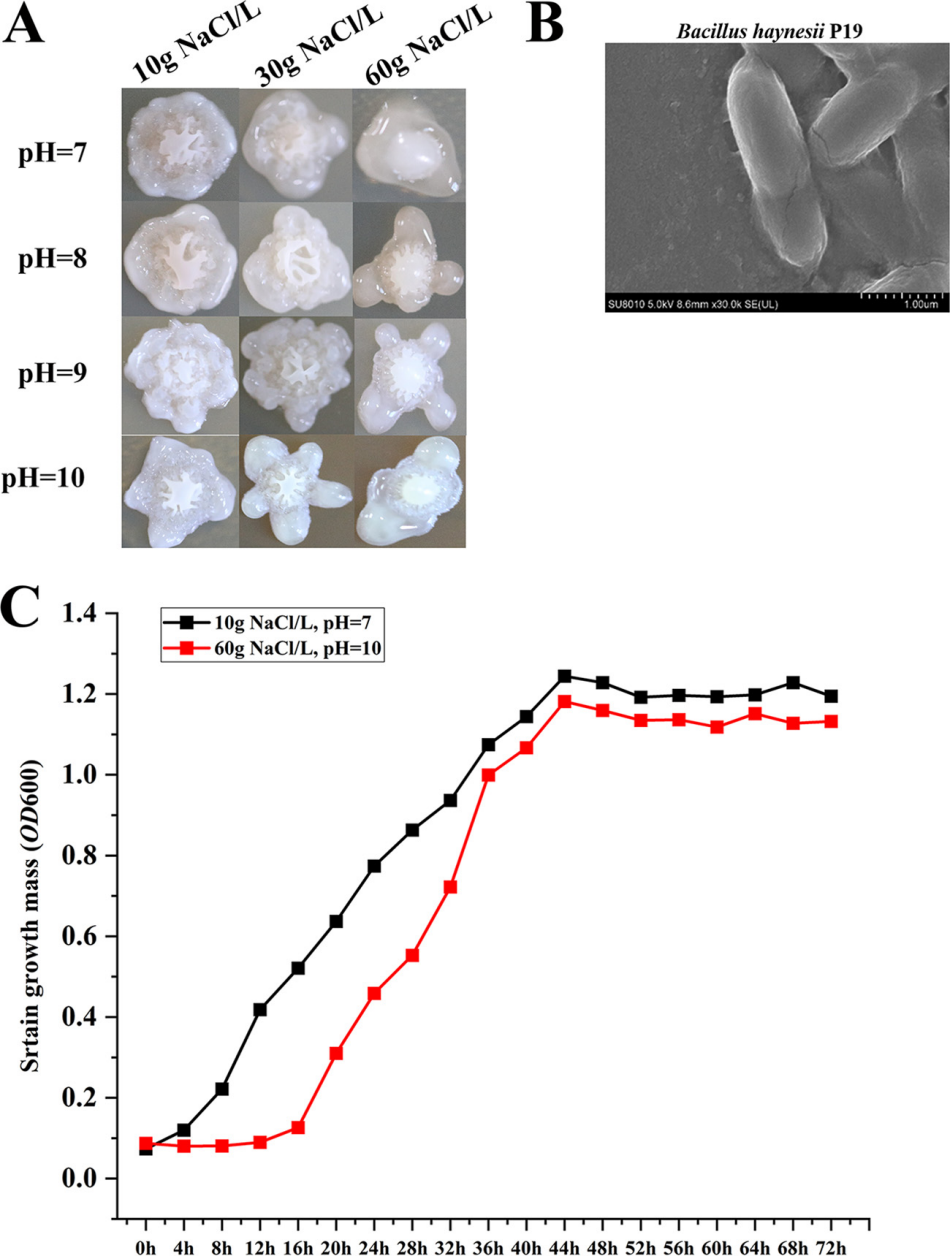
Salt-alkali tolerance of Bacillus haynesii P19. (A) Colony growth of Bacillus haynesii P19 under different salinity and alkalinity levels. (B) Scanning electron microscope image of Bacillus haynesii P19 in a normal environment. (C) Growth curve of Bacillus haynesii P19 under different salinity and alkalinity conditions.

As mentioned above, we screened a salt-tolerant strain, Bacillus haynesii P19, from the tissue of *P. euphratica*. In order to test its salt and alkali tolerance, we carried out liquid shake flask fermentation and solid plate spotting, and the results are shown in Fig. 6. Figure 6A shows the colony growth of Bacillus haynesii P19 on the LB solid plate under different salt concentrations and different pH conditions. It can be seen that the colony color was milky white, the shape was irregular, and the surface was raised with grooves. The colony could still grow under the conditions of 60 g/L NaCl and pH 10. Figure 6B shows the scanning electron microscope (SEM) image of Bacillus haynesii P19, whose cells are rod shaped and 2 to 3 μm in size. Figure 6C is the growth curve test of Bacillus haynesii P19 in normal LB liquid medium (pH 7, 10 g/L NaCl) and high-salinity LB liquid medium (pH 10, 60 g/L NaCl). It can be seen that under high pH and in a high-salt environment, Bacillus haynesii P19 took more time to adapt to the saline-alkali environment during the adaptation period, and when it reached the plateau period, the growth density of the bacteria was only slightly lower than that in the normal environment. Although Bacillus haynesii P19 could survive under the extreme condition of 10% NaCl and pH 11, its growth was poor, so it was not shown in Fig. 6. Since these results showed that Bacillus haynesii P19 had a high salt-alkali tolerance, we can try to use it as a chassis strain for research and industrial production.

### Conclusions.

In this study, we report the endogenous bacterial community structure of *P. euphratica* in different saline environments. Although the habitats were different, the compositions and structures of endophytic bacterial communities were similar, mainly including *Proteobacteria*, *Actinobacteria*, *Bacteroidetes*, and *Firmicutes*.

We compared the differences of microorganisms in different salinities; alpha diversity showed that the endogenous bacterial diversity of *P. euphratica* in different saline environments and different tissues was varied; in particular, the Shannon index and Chao index of the sap of *P. euphratica* under extreme salinity were lower than those in other groups. At the phylum level, in the endophytes of *P. euphratica*, the high-abundance *Firmicutes* under high salinity can form endospores to resist extreme conditions. The high-abundance *Deinococcus-Thermus* under moderate salinity were drought tolerant but not saline tolerant. At the genus level, the dominant genera of sap of *P. euphratica* under extreme salinity were very different from those of other groups, almost all of which were related to saline-alkali tolerance. The LEfSe analysis suggested that the endogenous bacteria in *P. euphratica* were significantly varied at the different taxonomic levels, indicating that the different growth environments of *P. euphratica* can affect the abundance of its endogenous bacteria. These clades that showed the advantage of abundance in the groups under extreme and very strong salinities not only were resistant to salt-alkali stress but also had certain advantages in other aspects: for example, *Cytophagaceae* are the main degraders of cellulosic polysaccharides.

By predicting the function of endophytic bacteria of *P. euphratica*, we found that the abundance of some enzymes and metabolic pathways of endophytes of *P. euphratica* increased with the increase of soil salinity. Most of the enzymes were related to energy metabolism and carbohydrate metabolism, such as the tricarboxylic acid cycle and glycolysis.

The saline-alkali-tolerant strain Bacillus haynesii P19 screened from the tissue of *P. euphratica* can still grow well under the conditions of 60 g/L NaCl and pH 10. Under these conditions, in addition to the adaptation period and the time to reach the plateau phase of the strain being longer than those in the normal environment, the bacterial growth density in the plateau phase was only slightly lower than that in the normal environment. This indicated that Bacillus haynesii P19 has great potential to be developed as an industrial fermentation chassis bacterium.

Taken together, this study has systematically described the endogenous bacterial flora of *P. euphratica* and compared the microbial divergences in different salinities. We found that different saline environments will affect the composition of the endogenous flora of *P. euphratica*. These findings may provide valuable information for understanding the relationship between the environment and endophytes of a plant. On the other hand, we found that the endophytes of *P. euphratica* may be involved in some of the metabolic pathways that tolerate salt stress. These provided evidence that the endogenous bacteria of the host plant had different expression mechanisms under different degrees of stress, and this mechanism was very obvious in the distribution of endophytes. However, due to the limitation of conditions, it was impossible to determine which specific metabolic pathways and physiological systems were regulated by the predicted microbial functions alone in a saline-alkali environment. Therefore, further exploration through metagenomics is needed in the future.

## MATERIALS AND METHODS

### Sample collection.

We collected four samples of *P. euphratica* xylem, three samples of *P. euphratica* sap, and two samples of rhizosphere soil from a *P. euphratica* forest (high-saline soil) in Zephyr, Xinjiang, China (E76°57.935′, N38°01.848′), and collected four samples of *P. euphratica* xylem, three samples of *P. euphratica* sap, and two rhizosphere soil samples as controls from a *P. euphratica* forest (mild saline soil) in Darbancheng, Xinjiang, China (E87°91.531′, N43°52.026′). To ensure the least damage to *Populus euphratica*, we used a scalpel to scrape 10 to 15 g of the lower xylem tissue and collected the sap from the incision of the tree trunk and soil samples at a depth of 5 cm in the corresponding area. The above samples were collected in October 2019. Since the location of the sample collection was in the *P. euphratica* forest reserve, in order to prevent the destruction of *P. euphratica* vegetation, we had no way to use traditional methods to collect samples, such as the five-point method, so we could select only typical samples to collect.

When collecting each sample, three parallel samples were taken and mixed into one sample, which was stored in a 20- by 20-cm sterile zip-lock bag and placed in an incubator with an ice bag for transportation to the laboratory. Prior to DNA extraction, samples were stored at −80°C.

### Determination of soil physicochemical and biological parameters.

We dried, crushed, and sieved (2-mm-pore-size sieve) the soil samples; estimated soil parameters with a soil-to-water ratio of 1:5 (wt/vol); and used a conductivity meter (DDSJ-318; Lei-Ci, China) to measure the total amount of water-soluble salt. The pH of the soil was measured with a pH meter (FE20; Mettler-Toledo Instruments, China). Soil salt content (grams per kilogram) was calculated as the total mass fraction of cations (K^+^, Na^+^, Ca^2+^, and Mg^2+^) and anions (CO_3_^2−^, HCO_3_^−^, Cl^−^, and SO_4_^2−^), in which K^+^ and Na^+^ were determined by flame photometry, Ca^2+^ and Mg^2+^ were determined by EDTA complex metric titration, CO_3_^2−^ and HCO_3_^−^ were determined by double indicator neutralization titration, Cl^−^ was determined by silver nitrate titration, and SO_4_^2−^ was determined by EDTA indirect titration. The specific determination method of each indicator refers to *Soil and Agricultural Chemistry Analysis* (41).

### DNA extraction and PCR amplification.

Total microbial DNA was extracted from all samples using the E.Z.N.A. soil DNA kit (Omega Bio-Tek, Norcross, GA, USA) according to manufacturer protocols. Subsequently, DNA concentration and purity were determined with a NanoDrop 2000 UV-visible (UV-vis) spectrophotometer (Thermo Fisher Scientific, Wilmington, DE, USA).

The hypervariable region V3-V4 of the bacterial 16S rRNA gene was amplified with primer pairs 338F (5′-ACTCCTACGGGAGGCAGCAG-3′) and 806R (5′-GGACTACHVGGGTWTCTAAT-3′) with an ABI GeneAmp 9700 PCR thermocycler (ABI, CA, USA) (42).

PCRs were performed in triplicate as follows: 4 μL of 5× FastPfu buffer, 2 μL of 2.5 mM deoxynucleoside triphosphates (dNTPs), 0.8 μL of each primer (5 μM), 0.4 μL FastPfu polymerase, 0.2 μL bovine serum albumin (BSA), 10 ng template DNA, and double-distilled water (ddH_2_O) were combined into a total volume of 20 μL. The PCR amplifications were performed as follows: 95°C for 3 min, followed by 30 cycles of 95°C for 30 s, 55°C for 30 s, and 72°C for 45 s, and a final extension of 72°C for 10 min. The PCR products (3 μL) were detected using 2% agarose gels containing ethidium bromide, purified with the AxyPrep DNA gel extraction kit (Axygen Biosciences, Union City, CA, USA), and quantified using QuantiFluor-ST (Promega, USA).

### Illumina MiSeq sequencing.

Purified amplicons were pooled in equimolar amounts and paired-end sequenced (2 × 300) on an Illumina MiSeq platform (Illumina, San Diego, CA, USA) according to standard protocols of Majorbio Bio-Pharm Technology Co., Ltd. (Shanghai, China).

### 16S rRNA gene sequence analysis.

The raw 16S rRNA gene sequencing reads were demultiplexed, quality filtered by Trimmomatic, and merged by FLASH with the indicated criteria. (i) The 300-bp reads were truncated at any site receiving an average quality score of <20 over a 50-bp sliding window, the truncated reads shorter than 50 bp were discarded, and reads containing ambiguous characters were also discarded. (ii) Only overlapping sequences longer than 10 bp were assembled according to their overlapped sequence. The maximum mismatch ratio of the overlapping region is 0.2. Reads that could not be assembled were discarded. (iii) Samples were distinguished according to the barcode and primers, and the sequence direction was adjusted; the barcode matching had to be exact, but a 2-nucleotide mismatch was allowed in primer matching.

### Statistical analysis.

We used Uparse (version 7.1) to analyze the data of the 16S rRNA gene. The obtained sequences were denoised, unified, and aligned into OTUs using definitions of ≥97% sequence similarity. The taxonomy of each OTU representative sequence was analyzed by RDP (Ribosomal Database Project) Classifier (v.2.11) against the 16S rRNA database (Silva SSU138) using the confidence threshold of 0.7.

At the same time, Mothur (v.1.30.2) was used to calculate the alpha diversity (including Shannon and Chao indexes) to reflect the species richness and diversity in the samples. Principal-coordinate analysis (PCoA) was done in R using the ape package from the Bray-Curtis distance matrix to analyze the beta diversity to compare the community compositions of the tested samples. R language (version 3.3.1) was used to analyze the endophytic bacterial composition of the samples.

In the LEfSe software (http://huttenhower.sph.harvard.edu/galaxy/root?tool_id=lefse_upload), the nonparametric Kruskal-Wallis (KW) rank sum test was first used to detect the differences in species abundance between the two groups and obtain significantly different species. Then, the Wilcoxon rank sum test was used to test the difference and consistency of the different species in the subgroups between different groups. Finally, LDA was used to estimate the impact of these different species on the difference between groups.

The PICRUSt2 tool (43) was used for functional gene annotation of the 16S rRNA amplicon sequences with reference to the KEGG (Kyoto Encyclopedia of Genes and Genomes) database. This approach was used to search for enzymes involved in functional KEGG pathways, which included biological reaction and regulation of gene expression associated with metabolism, genetic and environmental information processing, cellular processes, and human disease.

### Salt and alkali tolerance test of Bacillus haynesii P19.

LB liquid medium was prepared with different salt concentrations and different pH values (10 g/L peptone; 5 g/L yeast extract powder; 10 g/L, 30 g/L, and 60 g/L sodium chloride; pH 7.0, 8.0, 9.0, and 10.0), and LB solid medium was prepared with 19 g/L agar powder added to liquid medium. Bacillus haynesii P19 was inoculated into LB liquid medium with an inoculum of 3% and cultured for 72 h. The absorbance of the strain at 600 nm was measured with a microplate reader every 4 h, and Origin was used to plot the values. At the same time, Bacillus haynesii P19 was spotted on LB solid plates with different concentrations of NaCl and pH values, incubated at 37°C for 24 h, and photographed.

Then, the bacteria after centrifugal cleaning were smeared on the glass slide, dried naturally, fixed on the scanning electron microscope (SEM) copper plate with liquid conductive glue on the back side up, sprayed with gold, and then placed on the SEM stage to collect images.

### Data availability.

The sequence of *Bacillus haynesii* P19 has been deposited in GenBank under accession no. PRJNA648288.
